# Circulating metabolites from the choline pathway and acute coronary syndromes in a Chinese case-control study

**DOI:** 10.1186/s12986-020-00460-0

**Published:** 2020-05-24

**Authors:** Yuxiang Dai, Qianqian Tian, Jing Si, Zhonghan Sun, Shalaimaiti Shali, Lili Xu, Daoyuan Ren, Shufu Chang, Xin Dong, Hongxia Zhao, Zhendong Mei, Yan Zheng, Junbo Ge

**Affiliations:** 1 Department of Cardiology, Shanghai Institute of Cardiovascular Disease, ZhongShan Hospital, Fudan University, 1609 Xietu Road, Shanghai, 200032 China; 2 Department of Anthropology and Human Genetics, School of Life Sciences, Fudan University, Shanghai, China; 3 School of Kinesiology, Shanghai University of Sport, Shanghai, China; 4 Human Phenome Institute, Fudan University, 2005 Songhu Road, Shanghai, 200438 China; 5 Institute of translational medicine, Shanghai University, Shanghai, China

**Keywords:** Choline, Cardiovascular risk, Metabolite score, Trimethylamine N-oxide, Intestinal flora

## Abstract

**Background:**

Accumulating evidence shows that circulating levels of trimethylamine N-oxide, which is generated from the metabolism of dietary choline, may predict cardiovascular disease among Caucasians. Acute coronary syndrome (ACS), one common presentation of cardiovascular disease, is a spectrum of signs and symptoms due to acute decreased blood flow in the coronary arteries. The relationship between the metabolites from choline pathway and ACS remains unclear. We aimed to assess the associations of circulating metabolites from the choline pathway with ACS among a Chinese population, who consume a different dietary pattern than their Western counterparts.

**Methods:**

We recruited 501 participants who were admitted to the Department of Cardiology, Zhongshan Hospital,Shanghai China between March 2017 and June 2018, including 254 ACS cases and 247 controls. Liquid chromatography-tandem mass spectrometry was used to measure circulating concentrations of metabolites in the choline pathway, including betaine, choline, trimethylamine, and trimethylamine N-oxide. A composite metabolite score using a weighted sum of these four metabolites, and the betaine/choline ratio were calculated. Multivariable logistic regressions were applied to estimate the association of metabolites with ACS, with adjustment of age, sex, body mass index, smoking index, history of diseases, and kidney function.

**Results:**

After adjusting for traditional risk factors, per 1-standard deviation (SD) increment in choline was positively associated with the odds of ACS [odds ratio (OR), 95% confidence interval (CI), 1.77(1.44–2.18)], and the other metabolites were not associated with ACS at a statistical significance level. Compared with participants in the lowest quartile of the metabolite score, those in the highest quartile had higher odds of ACS [OR (95% CI), 3.18(1.85–5.54), *p* < 0.001 for trend]. Per 1-SD increment in metabolite score was positively associated with higher odds of ACS [OR (95% CI), 1.80 (1.37–2.40)], and per 1-SD increment in the betaine/choline ratio was inversely associated with the odds of ACS [OR (95% CI), 0.49 (0.39–0.60)].

**Conclusions:**

Among our Chinese participants, trimethylamine N-oxide was not associated with ACS, while a composite metabolite score of metabolites from the choline pathway was associated with increased odds of ACS. The choline pathway metabolites may be related to the pathophysiology of ACS among Chinese.

## Introduction

Acute coronary syndrome (ACS), a common subcategory of cardiovascular disease (CVD), has led to increased mortality globally [1]. ACS is a set of signs and symptoms due to acutely decreased blood flow in the coronary arteries, and the exact mechanism underlying its pathogenesis remains to be fully elucidated. Emerging metabolomics studies have provided novel pathways of CVD development, for example, intestinal microbiota related metabolites may play important roles in the progression of atherosclerosis [2, 3]. Remarkably, elevated levels of trimethylamine N-oxide (TMAO), a metabolite generated from the metabolism of dietary choline, carnitine and phosphatidylcholine (mostly originated from red meat, eggs, and fish) by gut microbiome [4], and its precursors were associated with increased risk of major adverse cardiovascular events in Western and European cohorts [5–7]. Thus far, little evidence exists regarding whether these metabolites are associated with ACS.

Different studies presented relatively heterogeneous results regarding metabolites from choline pathway and CVD [8, 9], and one possible reason could be different population structure and distinct dietary patterns [8]. Evidence from Chinese populations is in lack compared to the Caucasians. Moreover, most studies failed to assess the relationship between the combinded effect of circulating metabolites in the choline pathway and CVD. Hence, in this study, we sought to quantify four circulating metabolites from choline pathway including betaine, choline, trimethylamine (TMA) and TMAO, and to estimate the associations between choline pathway metabolites and ACS among Chinese participants.

## Methods

### Study population and biomedical measurements

A total of 501 participants were recruited from the Department of Cardiology, Zhongshan Hospital Shanghai China, between March 2017 and June 2018, including 254 cases and 247 controls. Cases were clinically diagnosed as ACS with documented ≥ 50% stenosis of at least one epicardial coronary artery during the coronary angiography [10, 11], and controls were non-ACS participants who underwent coronary angiography with a normal coronary artery. Each participant provided written informed consent. The study was approved by the Ethics Committee of the Zhongshan Hospital Fudan University.

Data on demographics (age and gender), anthropometrics (height and weight), lifestyle (smoking), history of disease (i.e., hypertension, diabetes mellitus and hyperlipidemia), kidney function [estimated glomerular filtration rate (eGFR)] were collected from the electronic medical record systems in Department of Cardiology. The severity of coronary atherosclerosis was assessed using the Gensini score, a widely used scoring system to quantify coronary atherosclerosis burden, in which a zero score indicates absence of atherosclerotic disease, and a higher score accounts for a severer proximal lesion by combining the degree of luminal narrowing as well as the location of narrowing [12, 13].

Fasting blood samples were collected using tubes containing EDTA via radial access before heparinization and then immediately stored at − 80 °C until analysis. Plasma proteins were precipitated with 3 volumes of methanol containing a mixed internal standard of 500 ng/ml. After vortex and centrifugation, supernatants were analyzed with an Agilent 1290 Infinity UHPLC instrument (Agilent, USA) on an XBridge BEH Hilic Column (2.5 μm,2.1 × 100 mm, Waters, Milford, MA) at a flow rate of 0.35 ml/min. LC gradient was starting from 2% 10 mM ammonium formate (A) and 98% acetonitrile of (B, PH3.5) over 1 min, then increased to 10% A at 6 min, holding 1 min; then to 15% A at 10 min, 30% A at 12 min and 40% A at 13 min. The supernatants of 20 samples were randomly taken out and mixed as Sample-Quality Control samples to calculate intra-day relative standard deviations and inter-day relative standard deviations, which were all < 15% in all the samples for the measured metabolites.

An Agilent 6470 Triple Quadruple (Agilent, USA) equipped with ESI source was used for quantification of TMA, TMAO, choline, and betaine. All the compounds were monitored in positive MRM mode using characteristic precursor-product ion transitions: m/z 60.1–44.2, m/z 76.1–58.1, m/z 104.1–60.1, m/z 118.1–58.1, m/z 162.1–102.9, m/z 114.0–44.1, respectively. The internal standards TMA-d9, TMAO-d9, and Choline-d9 were added to blood samples, and monitored in MRM mode at m/z69.1–49.2, m/z84.9–68.4, and m/z113.1–69.5, respectively. Series concentrations of TMA, TMAO, choline, and betaine standards and a fixed amount of internal standards were spiked into the water to prepare the curves for quantification of blood analytes. L-QC, M-QC and H-QC were inserted into the sequence to evaluate the accuracy of the method.

The standards for TMA, TMAO, choline and betaine were purchased from Sigma-Aldrich (Shanghai, China). The internal standards for TMA-d9 and TMAO-d9 were obtained from Cambridge Isotope Laboratories, Inc. (MA, USA).

### Statistical analyses

A rank-based inverse normal transformation was applied to approximate the normal distribution of metabolites concentrations [14]. Characteristics were presented as mean (SD) for continuous variables and number (frequencies) for categorical variables. Characteristics in cases and controls were compared using the *t*-test for continuous variables and χ^2^ -test for categorical variables. Cases were stratified into four groups (quartiles) according to their Gensini scores. We calculated a metabolite score as the weighted sum of levels of four metabolites from the choline pathway: betaine, choline, TMA and TMAO, and modeled the score as the main exposure variable in logistic regressions to estimate the composite association of circulating metabolites in choline pathway with ACS [15]. The weight for each metabolite was the regression coefficient for one SD increment in the blood concentration estimated from the adjusted multivariable logistic regression model. A ratio of betaine to choline was also calculated and modeled as an exposure variable in the regression models. This ratio can be considered as a better predictor of metabolic stress as it combines the predictive power of betaine and choline to metabolic stress together, and it was able to capture the composite associations of betaine and choline with metabolic disturbances [16].

Multivariable (adjusted) logistic regression models were used to evaluate the odds ratios (ORs) and corresponding 95% confidence intervals (CIs) to estimate the association of circulating metabolites and the metabolite score with the odds of ACS. Circulating metabolites were analyzed as both quartiles (using cut-points defined among controls) and continuous variables (per 1-SD increment). To test the linear trend across quartiles, the median of each quartile was assigned and analyzed as a continuous variable. Logistic regression models were adjusted for age, sex, smoking index (pack-years) and body mass index (BMI) in model 1, model 2 was additionally adjusted for history of the disease (ie., hypertension, diabetes mellitus and hyperlipidemia), and model 3 was further adjusted for kidney function. We used participants assigned to the first quartile of the levels of each metabolite, the metabolite score and betaine to choline ratio, as the reference group in each model. The correlations between circulating metabolites, metabolite score and betaine-to-choline ratio were tested by Spearman correlation. In ACS cases, the relations between circulating metabolites and the coronary atherosclerosis burden measured by Gensini score were also measured.

All statistical analyses and data visualizations were performed using R 3.6.2 (https://www.r-project.org/) and a two-sided *p* value< 0.05 was considered statistically significant.

## Results

### Characteristics of participants in ACS cases and controls

The characteristics of the study subjects by ACS status are presented in Table 1. This study included 430 (85.8%) men and 71 women, with a mean (SD) age of 62.92 (10.84) years. Compared with the controls, ACS cases were more likely to be current smokers (43.3%) and diabetes patients (28.0%) (*p* < 0.001), and the cases had a significantly higher BMI as expected. There were no significant differences between cases and controls concerning age, the proportions of gender, hypertension and hyperlipidemia as well as the eGFR levels (all *p* > 0.05).

**Table 1:** Characteristics of participants in cases of acute coronary syndrome and controls

Characteristics	Total (*n* = 501)	Cases (*n* = 254)	Controls (*n* = 247)	*p* value
Age, mean (SD), year	62.92 ± 10.84	63.07 ± 11.02	62.77 ± 10.69	0.54
Sex male, No. (%)	430(85.8%)	217(85.4%)	213(86.2%)	0.90
Current smokers, No. (%)	163(32.5%)	110(43.3%)	53(21.5%)	< 0.001
Hypertension, No. (%)	296(59.1%)	149(58.7%)	147(59.5%)	0.92
Diabetes mellitus, No. (%)	104(20.8%)	71(28.0%)	33(13.4%)	< 0.001
Hyperlipidaemia, No. (%)	13(2.6%)	5(2.0%)	8(3.2%)	0.54
BMI, mean (SD), kg/m^2^	25.06 ± 3.33	24.73 ± 3.53	25.41 ± 3.08	0.02
Smoking Index, mean (SD), pack-years	15.13 ± 25.21	17.96 ± 26.37	12.22 ± 23.73	< 0.001
eGFR, mean (SD), ml/min/1.73m^2^	82.36 ± 19.90	80.76 ± 20.79	84.01 ± 18.88	0.07
Gensini Score, mean (SD)	39.88 ± 52.01	76.73 ± 50.83	1.99 ± 2.90	< 0.001
Betaine, mean (SD), μmol/L	40.07 ± 15.94	41.74 ± 17.87	38.36 ± 13.55	0.02
Choline, mean (SD), μmol/L	47.93 ± 33.26	57.04 ± 34.70	38.55 ± 28.99	< 0.001
TMA, mean (SD), μmol/L	4.10 ± 2.99	4.27 ± 3.30	3.93 ± 2.63	0.20
TMAO, mean (SD), μmol/L	1.92 ± 2.08	1.84 ± 2.21	1.99 ± 1.95	0.42
Choline metabolite score, mean (SD)	34.58 ± 21.33	40.21 ± 22.39	28.78 ± 18.56	< 0.001
Ratio of betaine/choline, mean (SD), μmol/L	1.03 ± 0.44	0.89 ± 0.43	1.18 ± 0.40	< 0.05

*BMI* body mass index, *eGFR* estimated glomerular filtration rate, *TMA* trimethylamine, *TMAO* trimethylamine N-oxide

Choline metabolite score was applied by a weighted sum of concentrations of four metabolites in the choline pathway (betaine, choline, TMA and TMAO)

### Differences in circulating metabolites in the choline pathway between the ACS cases and controls

**Fig. 1:**
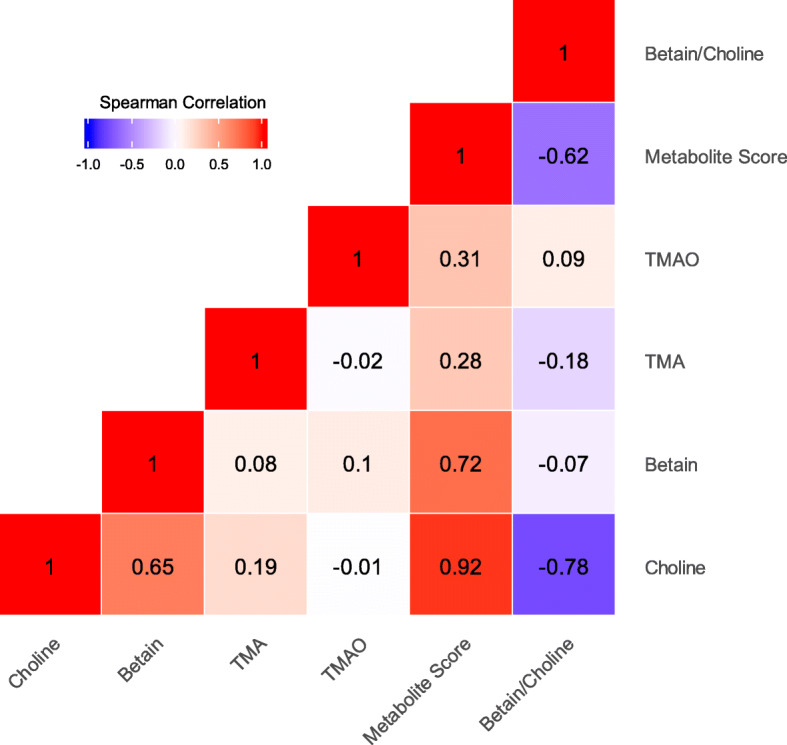
Spearman correlations of the choline pathway metabolites in all participants. Red represents a positive correlation, blue represents a negative correlation, and the number in the matrix is the coefficients of spearman correlation The inverse normal transformation was applied to the raw values of metabolites. To build the score, we applied a weighted sum of concentrations of 4 metabolites in the choline pathway (betaine, choline, TMA and TMAO). The ratio of betaine/choline was calculated by dividing the raw values and then applying the inverse normal transformations. TMA, trimethylamine; TMAO, trimethylamine N-oxide

In this study, ACS cases had significantly higher levels of betaine (mean ± SD, 41.74 ± 17.87 μmol/L in cases vs. 38.36 ± 13.55 μmol/L in controls), choline (57.04 ± 34.70 μmol/L in cases vs. 38.55 ± 28.99 μmol/L in controls) and metabolite score (40.21 ± 22.39 in cases vs. 28.78 ± 18.56 in controls) than controls (*p* < 0.05, Table 1). There were no significant differences in the levels of TMA and TMAO between cases and controls. A heatmap of Spearman correlation coefficients of the circulating metabolites analyzed in participants was shown in Fig. 1. Negative correlations were observed for choline and betaine-to-choline ratio, and metabolite score and betaine-to-choline ratio (*p* < 0.001).

### Circulating concentration of choline pathway metabolites with ACS

The associations of circulating concentrations of choline pathway metabolites and metabolite score with the odds of ACS were shown in Table 2. Compared with participants in the bottom quartile of choline, participants in the highest quartile of choline had significantly higher odds of ACS after adjusting for traditional risk factors [OR (95% CI), 3.72 (2.21–6.34), *p* < 0.001 for trend]. Per 1-SD increment concentrations of choline were associated with higher odds of ACS [OR (95% CI), 1.77(1.44–2.18)]. In multivariable-adjusted models, the choline metabolite score was associated with 3.18 fold higher odds of ACS across extreme quartiles [OR (95 CI%), 3.18 (1.85–5.54), *p* < 0.001 for trend]. Per 1-SD increment in choline metabolite score was associated with higher odds of ACS [OR (95 CI%), 1.80(1.37–2.40)], and that in betaine-to-choline ratio was inversely associated with the odds of ACS [OR (95 CI%), 0.49 (0.39–0.60)]. Neither TMA nor TMAO was associated with ACS status in our population.

**Table 2:** Odds of acute coronary syndrome by circulating concentration of choline pathway metabolites

Variable	Odds Ratio (95% CI) for Quartiles of Metabolites Concentration				*P* for trend	Odds Ratio (95% CI) per 1-SD increment
	1	2	3	4		
**Betaine**						
No. of controls	62	62	61	62	…	…
No. of cases	59	45	53	97	…	…
Model 1 ^a^	Ref.	0.69 (0.40–1.18)	0.84 (0.50–1.43)	1.43 (0.87–2.34)	0.07	1.11(0.93–1.33)
Model 2 ^b^	Ref.	0.62 (0.35–1.07)	0.76 (0.44–1.31)	1.43 (0.87–2.36)	0.07	1.12(0.93–1.35)
Model 3 ^c^	Ref.	0.63 (0.36–1.09)	0.78 (0.45–1.34)	1.41 (0.85–2.34)	0.08	1.12(0.93–1.34)
**Choline**						
No. of controls	62	62	61	62	…	…
No. of cases	35	28	27	164	…	…
Model 1 ^a^	Ref.	0.82 (0.44–1.53)	0.70 (0.37–1.31)	4.36 (2.61–7.38)	< 0.001	1.85(1.52–2.28)
Model 2 ^b^	Ref.	0.79 (0.42–1.47)	0.58 (0.30–1.11)	3.91 (2.33–6.65)	< 0.001	1.79(1.47–2.21)
Model 3 ^c^	Ref.	0.73 (0.39–1.39)	0.55 (0.29–1.06)	3.72 (2.21–6.34)	< 0.001	1.77(1.44–2.18)
**TMA**						
No. of controls	62	62	61	62	…	…
No. of cases	70	49	59	76	…	…
Model 1 ^a^	Ref.	1.41 (0.85–2.34)	0.94 (0.56–1.58)	0.67 (0.39–1.15)	0.02	1.11(0.92–1.34)
Model 2 ^b^	Ref.	1.53 (0.92–2.57)	1.09 (0.64–1.85)	0.76 (0.44–1.32)	0.07	1.05(0.87–1.27)
Model 3 ^c^	Ref.	1.71 (1.01–2.91)	1.26 (0.73–2.17)	0.80 (0.46–1.40)	0.09	1.10(0.90–1.33)
**TMAO**						
No. of controls	62	62	61	62	…	…
No. of cases	85	62	42	65	…	…
Model 1 ^a^	Ref.	0.79 (0.47–1.35)	0.95 (0.57–1.60)	1.25 (0.75–2.08)	0.37	0.87(0.72–1.05)
Model 2 ^b^	Ref.	0.70 (0.40–1.20)	0.91 (0.54–1.53)	1.01 (0.60–1.70)	0.76	0.84(0.69–1.02)
Model 3 ^c^	Ref.	0.71 (0.41–1.23)	0.94 (0.56–1.59)	1.09 (0.64–1.85)	0.56	0.80(0.65–0.97)
**Metabolite Score**						
No. of controls	62	62	61	62	…	…
No. of cases	41	26	59	128	…	…
Model 1 ^a^	Ref.	1.10(0.60–2.03)	1.65(0.93–2.97)	3.65(2.14–6.32)	< 0.001	1.96(1.49–2.59)
Model 2 ^b^	Ref.	1.00(0.54–1.86)	1.39(0.77–2.52)	3.26(1.90–5.67)	< 0.001	1.85(1.40–2.45)
Model 3 ^c^	Ref.	1.00(0.54–1.86)	1.35(0.75–2.46)	3.18(1.85–5.54)	< 0.001	1.80(1.37–2.40)
**Betaine/choline ratio**						
No. of controls	62	62	61	62	…	…
No. of cases	160	39	24	31	…	…
Model 1 ^a^	Ref.	0.23(0.14–0.39)	0.16(0.09–0.29)	0.19(0.11–0.31)	< 0.001	0.46(0.37–0.57)
Model 2 ^b^	Ref.	0.24(0.14–0.39)	0.17(0.09–0.30)	0.20(0.12–0.35)	< 0.001	0.48(0.39–0.59)
Model 3 ^c^	Ref.	0.24(0.14–0.41)	0.17(0.09–0.30)	0.21(0.12–0.36)	< 0.001	0.49(0.39–0.60)

The inverse normal transformation was applied to the raw values of metabolites. To build the score, we applied a weighted sum of concentrations of 4 metabolites in the choline pathway (betaine, choline, TMA, and TMAO). The ratio of betaine/choline was calculated by dividing the raw values and then applying the inverse normal transformations

*TMA* trimethylamine, *TMAO* trimethylamine N-oxide. Ref., i.e., reference group, and we used participants assigned to the first quartile of the concentrations of each metabolite, the metabolite score and betaine to choline ratio, as the reference group in each model

^a^ in model 1, odds ratio was adjusted for age, sex, smoking index and body mass index;

^b^ in model 2, odds ratio was adjusted for all factors in model 1, plus history of the disease (i.e., hypertension, diabetes mellitus and hyperlipidemia);

^c^ in model 3, odds ratio was adjusted for all factors in model 2, plus kidney function measured by eGFR

### Relations between Gensini score and circulating metabolites in ACS cases

The associations of circulating concentrations of choline pathway metabolites and metabolite score with Gensini score in ACS cases were shown in Table 3**.**Based on our results, no significant association was observed between circulating metabolites and Gensini score among ACS cases.

**Table 3:** Relations between Gensini score and circulating metabolites in acute coronary syndrome patients

Variable	Adjusted means (standard errors) of Gesini score across quartiles of metabolites levesl				*P* for trend	Regression coefficient (95% CI) per 1-SD increment ^d^
	1	2	3	4		
**Betaine**						
Model 1 ^a^	75.7 (7.68)	75.0 (8.33)	73.7 (7.75)	71.1 (6.37)	0.57	-0.18 (-6.12~5.75)
Model 2 ^b^	71.8 (13.7)	72.0 (13.5)	68.9 (14.0)	68.4(12.9)	0.65	0.11 (-5.82~6.04)
Model 3 ^c^	71.8 (13.7)	72.0 (13.6)	68.9 (14.0)	68.4 (12.9)	0.65	0.11 (-5.84~6.06)
**Choline**						
Model 1 ^a^	71.6 (9.44)	73.1 (10.34)	62.4 (10.42)	75.7 (5.29)	0.50	4.32 (-2.03~10.66)
Model 2 ^b^	69.8 (14.6)	72.0 (14.4)	54.8 (15.7)	71.5 (12.5)	0.68	3.77 (-2.63~10.16)
Model 3 ^c^	69.8 (14.7)	72.0 (14.4)	54.8 (15.7)	71.5 (12.6)	0.68	3.77 (-2.64~10.19)
**TMA**						
Model 1 ^a^	77.0 (7.44)	77.8 (8.20)	72.7 (7.57)	69.3 (6.63)	0.30	-3.78 (-10.08~2.52)
Model 2 ^b^	78.2 (13.7)	74.8 (13.8)	72.9 (13.6)	63.6 (12.8)	0.11	-5.40 (-11.85~1.06)
Model 3 ^c^	78.3 (13.7)	74.7 (13.8)	72.8 (13.7)	63.5 (12.9)	0.11	-5.56 (-12.13~1.00)
**TMAO**						
Model 1 ^a^	73.0 (6.47)	79.0 (7.59)	66.8 (8.68)	73.4 (7.36)	0.80	-1.28 (-7.84~5.27)
Model 2 ^b^	67.3 (13.4)	76.0 (13.3)	62.5 (14.5)	68.9 (14.5)	0.92	-1.17 (-7.84~5.51)
Model 3 ^c^	67.5 (13.5)	76.2 (13.4)	62.6 (14.6)	68.8 (13.0)	0.92	-1.29 (-8.33~5.75)
**Metabolite Score**						
Model 1 ^a^	76.7 (9.82)	63.0 (9.01)	73.4 (7.46)	76.2 (5.87)	0.55	3.14 (-7.23~13.51)
Model 2 ^b^	75.4 (14.8)	61.5 (14.2)	68.4 (13.4)	73.5 (12.7)	0.64	2.46 (-7.93~12.85)
Model 3 ^c^	75.3 (14.8)	61.3 (14.3)	68.4 (13.5)	73.5 (12.7)	0.64	2.49 (-7.99~12.96)
**Ratio of betaine/choline**						
Model 1 ^a^	76.2 (5.33)	71.1 (8.55)	79.7 (11.19)	58.4 (9.83)	0.15	-4.9 (-11.21~1.41)
Model 2 ^b^	73.7 (13.0)	68.7 (13.3)	78.9 (15.6)	58.4 (15.0)	0.26	-4.15 (-10.55~2.25)
Model 3 ^c^	73.7 (13.0)	68.5 (13.4)	78.8 (15.7)	58.4 (15.0)	0.26	-4.15 (-10.56~2.26)

Inverse normal transformation was applied to raw values of metabolites. To build the score, we applied a weighted sum of concentrations of 4 metabolites in the choline pathway (betaine, choline, TMA and TMAO). The ratio of betaine/choline was calculated by dividing the raw values and then applying inverse normal transformations

TMA, trimethylamine; TMAO, trimethylamine N-oxide

^a^ Model 1 was adjusted for age, sex, smoking index and body mass index

^b^ Model 2 was adjusted for all factors in model 1, plus history of disease (i.e., hypertension, diabetes mellitus and hyperlipidemia)

^c^ Model 3 was adjusted for all factors in model 2, plus kidney function measured by eGFR

^d^ The regression coefficient represented the average difference in the Gensini score per 1-SD increase in the transformed levels of metabolites

## Discussion

In this study of Chinese participants, we observed that higher levels of single metabolite choline, as well as a composite metabolite score representing the comprehensive effect of circulating metabolites in choline pathway, were associated with higher odds of ACS. The betaine-to-choline ratio was inversely associated with the odds of ACS. To our knowledge, this was the first study to evaluate the potential association between circulating microbial metabolites in choline pathway and ACS in Chinese population, and may provide novel perspectives on the choline metabolites and CVD.

It has long been known that habitual dietary pattern is a significant contributor to the CVD risk [17]. Gut microbial metabolism of dietary nutrients may result in the production of proatherogenic circulating factors that act through a meta-organismal endocrine axis to impact CVD risk [18]. For example, circulating metabolites in the choline pathway are derivatives from dietary nutrients, and the gut microbiome is actively involved in its metabolism pathway [19]. Our findings suggest that circulating metabolites in the choline pathway may play a role in the ACS pathophysiology in Chinese, reflecting a link between dietary metabolism, gut microbiome and ACS development. Several mechanisms, such as pro-atherogenic and pro-thrombotic effects can at least partially explain these associations between metabolites and ACS [2, 7].

There is accumulating evidence of the associations between TMAO and cardiovascular events, and a meta-analysis of 19 cohorts suggested a positive association of TMAO and its precursors with the risk of major adverse cardiovascular endpoints [8]. However, it should be noted that most cohorts were conducted in western populations, while the western diet patterns contain abundant ﻿nutrient precursors for TMAO [7]. Choline-rich foods (such as red meat, egg yolks, and milk) are converted by the gut microbiota into TMA, which is further oxidized into TMAO in the liver by the hepatic flavin-containing monooxygenase family of enzymes [20]. Chronic consumption of diets rich in red meat increased both plasma and urine TMAO levels [21]. In this study of a Chinese population, the median of TMAO levels was 1.45 μmol/L (interquartile range: 0.94–2.32), which was lower than that in western populations [7, 15, 22], and similar to that in another study conducted in Chinese populations [23].

Previous studies in western populations have shown that TMAO levels were related to atherosclerosis and CVD, and reducing TMAO production may be a potential therapeutic target for CVD [2, 3, 24]. However, the association between TMAO and adverse outcomes was distinctive in different races [25]. Moreover, a recent bi-directional Mendelian randomization analysis suggested that TMAO levels were elevated in patients of type 2 diabetes and chronic kidney disease, and that confounding or reverse causality should also be considered when explaining the previously reported associations [26]. In the current study, we did not found a significant association between circulating TMAO concentration and ACS. Factors such as the differences in dietary choline intake, intestinal microbiota activity and flavin monooxygenase activity, and the divergent concentrations of TMAO in different populations as mentioned above, may explain the null association between TMAO and ACS through complex mechanisms in our Chinese population.

As the metabolic precursors of TMAO, choline (the precursor of betaine) and betaine have also been linked with the risk of CVD in several studies [15, 27–30]. The major fate of dietary choline is conversion to phosphatidylcholine, which is essential in the secretion of very-low-density lipoproteins from the liver [31]. Betaine is a determinant for homocysteine concentration, which may increase abnormal platelet activity and blood coagulation, leading to thrombotic disorders such as ACS [32]. In an ACS cohort study, plasma betaine was found negatively correlated with triglyceride and non-high-density lipoprotein cholesterol and low plasma betaine concentrations contributed to metabolic syndrome and known prognostic markers of vascular diseases [29, 30]. However, a systematic review and meta-analysis of six prospective studies from three countries (USA, Japan, and The Netherlands) did not suggest the associations of choline or betaine with incident CVD or CVD mortality [27]. In our study, we did not find a statistically significant association between blood betaine levels and ACS, although the betaine/choline ratio was inversely associated with the odds of ACS. Our results in a Chinese population are consistent with that in a case-control study conducted within the PREDIMED (Prevention With Mediterranean Diet) trial in the Spanish populations [15]. Because the betaine/choline ratio combines the predictive power of the choline (precursor) and betaine (product) pathway, it may further state the importance of the choline pathway in the ACS pathogenesis. Platelet activity, blood coagulation and lipid profile may be the underlying mechanisms [32].

Several limitations of this analysis are noteworthy. First, the causality of the observed relationship cannot be inferred because of the case-control study design. In particular, residual confounding such as medication use cannot be excluded. Second, the participants were patients who visited Zhongshan hospital and were mostly unhealthy in general. Thus, our results might not be generalizable to the general Chinese population. Third, we did not collect dietary information or fecal samples for each participant, and therefore could not explore the complete association among dietary intake, gut microbiome and circulating metabolites.

## Conclusion

The present study suggested that circulating choline levels were higher in patients with ACS among Chinese participants. A higher composite metabolite score of metabolites from the choline pathway, which were related to the gut microbiome, was associated with increased odds of ACS in Chinese participants. The choline pathway may be related to the pathophysiology of ACS, and further mechanistic studies are warranted.

## Data Availability

The datasets used and/or analysed during the current study are available from the corresponding authors on reasonable request.

## Abbreviations

ACS: Acute coronary syndrome
CVD: Cardiovascular disease
TMAO: Trimethylamine N-oxide
TMA: Trimethylamine
eGFR: Estimated glomerular filtration rate
LC-MS: Liquid chromatography tandem mass spectrometry
OR: Odds ratio
CI: Confidence interval BMI Body mass index
